# Comparison of coronary artery bypass grafting and percutaneous coronary intervention in patients with heart failure with reduced ejection fraction and multivessel coronary artery disease

**DOI:** 10.18632/oncotarget.25006

**Published:** 2018-04-20

**Authors:** Michał Hawranek, Michal O. Zembala, Mariusz Gasior, Tomasz Hrapkowicz, Łukasz Pyka, Daniel Cieśla, Marian Zembala

**Affiliations:** ^1^ 3rd Department of Cardiology, School of Medicine with the Division of Dentistry in Zabrze, Medical University of Silesia, Katowice, Silesian Centre for Heart Disease, Zabrze, Poland; ^2^ Department of Cardiac, Vascular and Endovascular Surgery and Transplantology, School of Medicine with the Division of Dentistry in Zabrze, Medical University of Silesia in Katowice, Silesian Center for Heart Diseases, Zabrze, Poland; ^3^ Department of Science, Biostatistics and New Technologies, Silesian Centre for Heart Disease, Zabrze, Poland

**Keywords:** chronic heart failure, ischemic cardiomyopathy, percutaneous coronary interventions, coronary artery bypass grafting, revascularization

## Abstract

**Aims:**

To compare coronary artery bypass grafting (CABG) with percutaneous coronary interventions (PCI) in patients with heart failure with reduced ejection fraction (HFrEF) and multivessel coronary artery disease.

**Methods:**

1213 patients were selected from institutional databases, 761 and 452 in CABG and PCI group respectively. Only the subjects with left ventricle ejection fraction ≤ 35% and multivessel coronary artery disease were included to the study. The primary outcome measure was long-term all-cause death, the secondary outcomes were recurrent myocardial infarction, urgent repeat revascularization and stroke. Propensity Score-Based Adjusted Survival Curves were used for revascularization methods comparison.

**Results:**

Survival rates were similar in both groups (HR, 0.91; 95% CI, 0.65-1.28; p=0.59). Recurrent myocardial infarction was observed significantly less often in the CABG group (HR, 0.44; 95% CI, 0.26-0.74; p=0.002). Repeat urgent revascularization was less frequent in the CABG group (HR, 0.50; 95% CI, 0.30-0.84; p=0.008). The rate of stroke did not differ between the groups (HR, 1.17; 95% CI, 0.62-2.22; p=0.62).

**Conclusions:**

In patients with HFrEF and multivessel CAD revascularization both with CABG and PCI resulted in similar survival rates. PCI is associated with increased risk of recurrent MI and urgent repeat revascularization, whereas the risk of stroke is similar in both methods.

## INTRODUCTION

The estimated population incidence of heart failure (HF) in the developed countries ranges from 1% to 2% and at least one-half have heart failure with reduced ejection fraction (HFrEF) [1, 2]. Coronary artery disease (CAD) is the most common etiology for HFrEF and has increased with the growing incidence of associated mortality [2]. This unfavorable prognosis is related to progressive nature of ischemic left ventricle (LV) dysfunction and concomitant comorbidities, such as chronic kidney disease or diabetes.

Guideline-directed medical therapy remains the most important option and is associated with undisputed benefit in survival and quality of life [2]. Revascularization may have an additive positive effect on prognosis [3]. In the recent guidelines on myocardial revascularization, coronary artery bypass grafting (CABG) has a class I recommendation, whereas percutaneous coronary interventions (PCI) only class IIb [4]. However, in everyday clinical practice many patients with HFrEF and multivessel CAD are treated with PCI. The advantage of CABG over PCI is based on the clinical trials comparing CABG with medical treatment, which reported survival benefit in this population of patients [5]. There are no such trials regarding PCI. Moreover, in last decades the only study dedicated to compare these two revascularization methods in HFrEF population was finished prematurely [6].

Additionally, trials comparing CABG vs PCI in stable CAD rarely included patients with HFrEF [7, 8]. On the other hand, PCI in patients with HFrEF and CAD might be a reasonable alternative for surgical treatment. Decreased LVEF is one of the predictors of poor outcomes after CABG [9]. Moreover, HF patients have significant morbidity, which additionally increases the risk of intervention. Therefore, having at our disposal a large population of patients with HFrEF of ischemic etiology treated with either CABG or PCI, we aimed to compare these revascularization methods to assess their impact on prognosis.

## RESULTS

**Figure 1:**
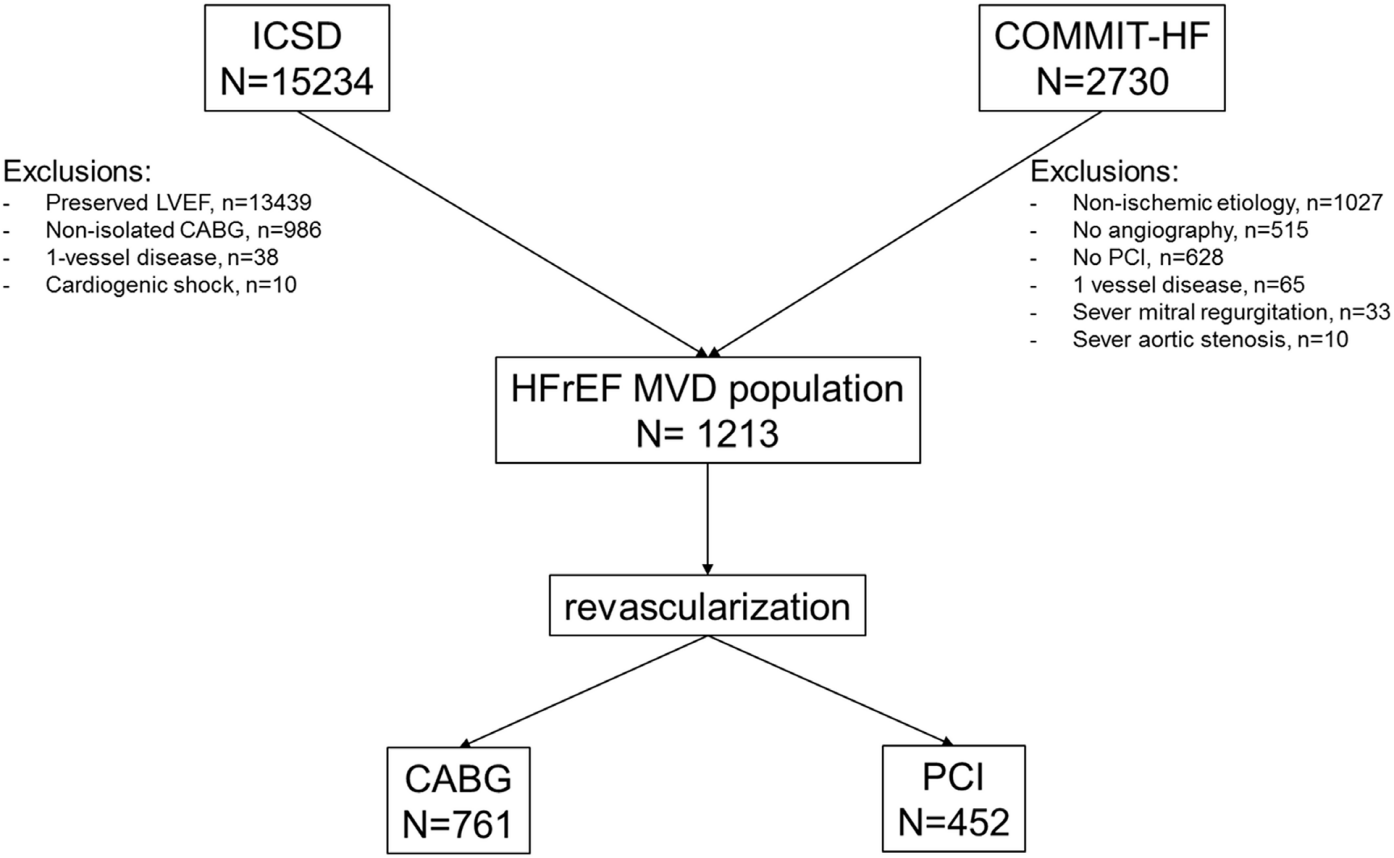
Study flow chart MVD, multivessel coronary artery disease; COMMIT-HF, COnteMporary Modalities in Treatment of Heart Failure Registry; HFrEF, heart failure with reduced ejection fraction; ICSD, institutional cardiac surgery database; PCI, percutaneous coronary interventions; CABG, coronary artery bypass grafting; LVEF, left ventricle ejection fraction.

Among 2730 patients in COMMIT-HF Registry and 15234 in ICSD, 1217 were finally included into analysis, 761 and 452 in the CABG and PCI group respectively (Figure 1). Most patients were excluded due to non-performance of invasive diagnostics and therapy (n=1143) and non-ischemic etiology of HF (n=1027) in the PCI group. In the CABG patients, preserved LVEF (n=13439) and non-isolated CABG (n=986) were the most common exclusions.

Baseline characteristic is shown in Table 1. The PCI group had worse clinical profile with higher incidence of typical risk factors, prior ischemic events and prior revascularization procedures. New York Heart Association (NYHA) class III and IV were observed more frequently in this group of patients. The mean LV ejection fraction was significantly lower in the PCI group. These differences were reflected in the Euroscore II risk scale, which was significantly higher in patients treated with PCI.

**Table 1:** Baseline characteristics

Factor	Study population	N = 1213	P value
	CABG N = 761	PCI N = 452	
Age, years ± SD	64.7 ± 9.0	65.3 ± 10.2	0.32
Male, %	82.5	84.1	0.54
BMI, kg/m^2^ ± SD	27.7 ± 4.4	28.3 ± 4.4	0.07
Arterial hypertension, %	79.2	63.1	<0.001
Prior one MI, %	54.9	53.5	0.68
Prior two or more MI, %	20.6	30.1	<0.001
Prior PCI, %	37.6	62.8	<0.001
Prior CABG, %	0.5	20.6	<0.001
Atrial fibrillation, %	11.6	23.0	<0.001
Prior stroke, %	9.1	7.3	0.33
Diabetes mellitus, %	37.3	47.8	<0.001
Dyslipidemia, %	62.0	48.0	<0.001
COPD, %	13.4	10.0	0.09
Neoplastic disease, %	16.5	22.0	0.03
Malignant neoplasms, %	9.5	12.2	0.18
Benign neoplasms, %	7.0	9.7	0.14
NYHA Class^*^			
I, %	21.3	16.6	0.06
II, %	59.9	38.3	<0.001
III, %	17.1	36.3	<0.001
IV, %	1.7	8.9	<0.001
eGFR^*^, 30-60 ml/min/1.73m^2^, %	11.3	12.6	0.55
eGFR^*^, < 30 ml/min/1.73m^2^, %	7.4	10.4	0. 08
LVEF^*^, % ± SD	30.9 ± 4.5	27.4 ± 5.5	<0.001
EUROSCORE 2 scale, %	3.64 ± 4.36	4.49 ± 4.86	0.001

BMI, body mass index; CABG, coronary artery bypass grafting; CAD, coronary artery disease; COPD, chronic obstructive pulmonary disease; eGFR = estimated glomerular filtration rate; LVEF, left ventricular ejection fraction; MI, myocardial infarction; NYHA, New York Heart Association; PCI, percutaneous coronary intervention; Q1-Q3, quartile 1 and quartile 3; SD, standard deviation.

All the patients included into analysis had at least two major coronary arteries diseased with higher prevalence of 3-vessel disease in the CABG group. Moreover, left main (LM) disease, left coronary artery disease and chronic total occlusion occurrence were found more often in this group. These data confirm more complex atherosclerosis in surgically treated patients. Angiographic and procedural characteristics are shown in Table 2. All the patients treated with CABG had at least one arterial graft, typically left internal mammary artery to the left anterior descending artery (86.7%). During PCI procedures, drug eluting stents (DES) were used more frequently than bare metal stents (BMS). Complete anatomical revascularization was achieved significantly more often in the CABG group. A detailed analysis of patients treated with CABG or PCI with DES only is provided in the supplementary materials. (Supplementary Tables 1–3 and Supplementary Figures 6–9).

**Table 2:** Angiographic and procedural characteristics

Factor	Study population	N = 1213	P value
	CABG N = 761	PCI N = 42	
2-vessel disease, %	24.8	48.0	<0.001
3-vessel disease, %	75.2	52.0	<0.001
Territory			
LM, %	38.5	13.3	<0.001
LAD/D1, %	99.5	91.8	<0.001
Cx/OM/IM, %	91.7	80.1	<0.001
RCA/PDA, %	84.0	80.1	0.1
CTO, %	56.5	39.6	<0.001
CTO, mean ± SD	0.85 ± 0.91	0.51 ± 0.73	<0.001
No of grafts, mean ± SD	2.50 ± 0.93		
- Arterial grafts, mean ± SD	1.01 ± 0.50		
- Saphenous grafts, mean ± SD	1.49 ± 0.97		
No of stents, mean ± SD		1.75 ± 1.06	
- DES, mean ± SD		1.29 ± 1.19	
- BMS, mean ± SD		0.47 ± 0.90	
Complete revascularization, %	68.6	54.0	<0.001

BMS, bare metal stents; CABG, coronary artery bypass grafting; CTO, chronic total occlusion; Cx, circumflex artery; D1, first diagonal branch; DES, drug eluting stents; IM, intermediate branch; LAD, left anterior descending; LM, lef t main; OM, obtus marginal branch; PDA, posterior descending artery; PCI, percutaneous coronary intervention; RCA, right coronary artery; SD, standard deviation.

### Short-term outcomes

In hospital, 30-day and 1-year outcomes were shown in Table 3. There were no differences in the prognosis besides the higher incidence of myocardial infarction (MI) in the PCI group at one year. The rate of stroke was low within the first 30 days after the procedure even in the surgical group with similar percentage in both groups after 1 year.

**Table 3:** In-hospital, 30 day and 1 year outcomes

Factor	Study population	N = 1213	P value
	CABG N = 761	PCI N = 452	
In-hospital			
death, %	1.6	0.9	0.45
MI, %	0.9	1.3	0.7
stroke, %	0.8	0.4	0.72
30 day			
Death, %	3.0	2.4	0.67
MI, %	1.5	2.2	0.45
Stroke, %	1.1	0.4	0.42
1 year			
Death, %	9.7	10.2	0.87
MI, %	2.0	5.9	0.002
Stoke, %	2.3	2.5	0.97

Abbreviations: CABG, coronary artery bypass grafting; MI, myocardial infarction; PCI, percutaneus coronary intervention.

### Long-term outcomes

**Figure 2:**
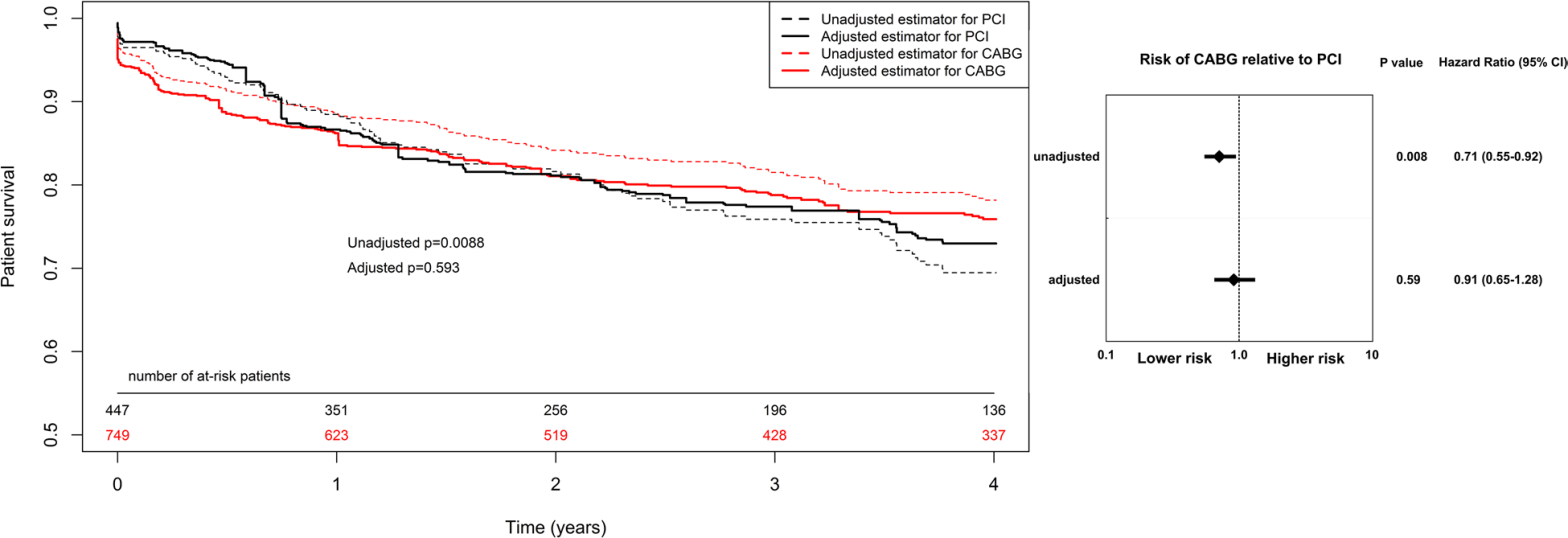
Kaplan–Meier curves and Forest plot for long term all-cause death CABG, coronary artery bypass grafting; CI, confidential interval; HR, hazard ratio PCI, percutaneous coronary interventions. Results adjusted for: sex, age, diabetes mellitus, hypertension, New York Heart Association classification at admission, Left Ventricle Ejection Fraction, previous stroke, previous transient ischaemic attack, chronic obstructive pulmonary disease, atrial fibrillation, chronic kidney disease, previous PCI, left main disease, two and three vessel coronary artery disease.

**Figure 3:**
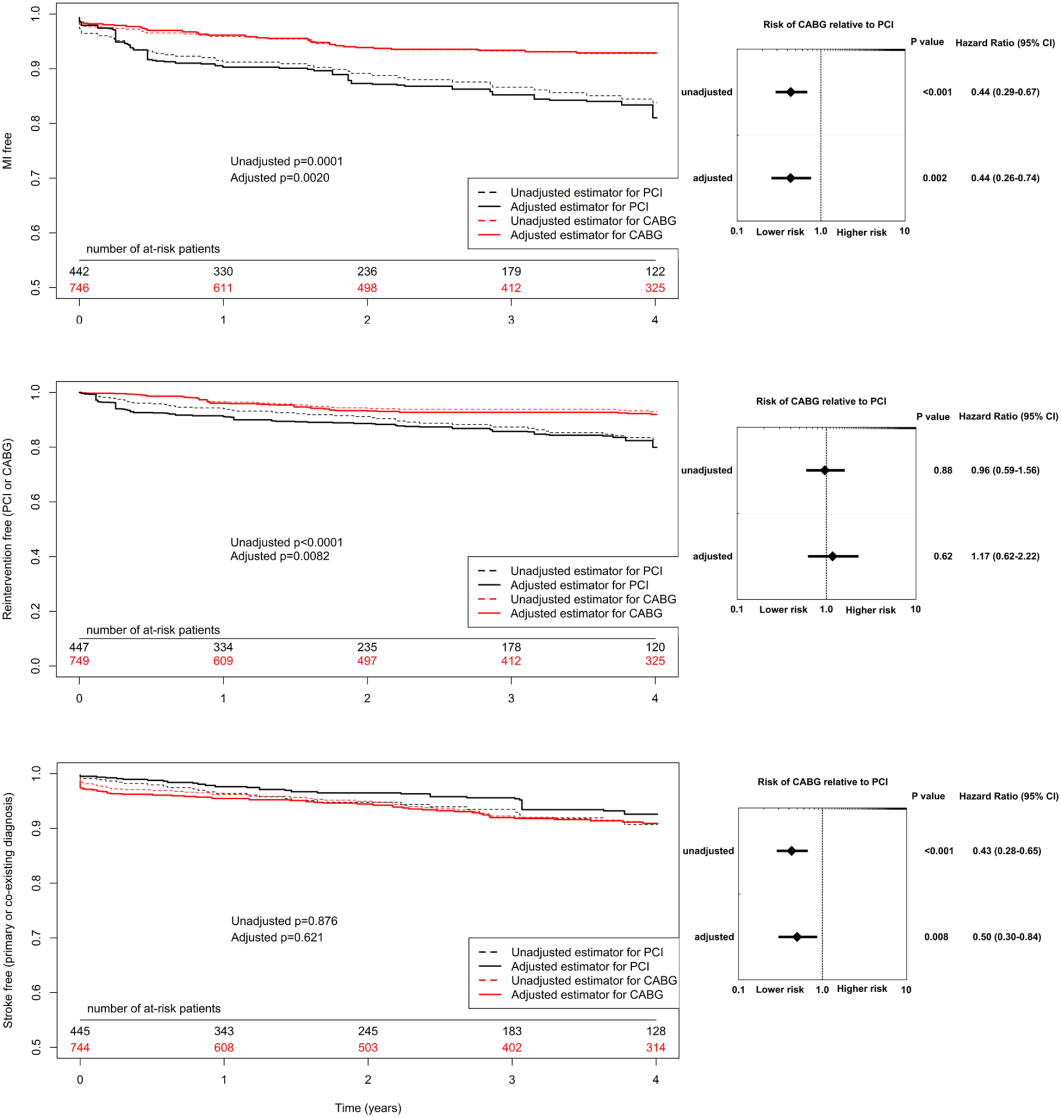
Kaplan–Meier curves and Forest plot for long term secondary outcomes (A, myocardial infarction; B, urgent revascularization; C, stroke) CABG, coronary artery bypass grafting; CI, confidential interval; HR, hazard ratio PCI, percutaneous coronary interventions. Results adjusted for: year of intervention, male sex, age, diabetes mellitus, hypertension, New York Heart Association classification at admission, left ventricle ejection fraction, previous stroke, previous transient ischaemic attack, chronic obstructive pulmonary disease, atrial fibrillation, chronic kidney disease, previous PCI, left main disease, two- and three vessel coronary artery disease.

The primary outcome measure was all-cause long-term rate of death. Kaplan–Meier curves are presented in Figure 2. Death occurred less often in the CABG patients (HR, 0.71; 95% CI, 0.55-0.92; p=0.008), however after adjustment for clinical and angiographic differences, survival rates were similar in both groups (HR, 0.91; 95% CI, 0.65-1.28; p=0.59). Results of secondary outcomes are shown in Figure 3. Recurrent MI was observed significantly less often in the CABG group (HR, 0.44; 95% CI, 0.26-0.74; p=0.002). More than 50% of the events occurred within the first year after index procedure. Similarly, repeat urgent revascularization was less frequent in the CABG group regardless of the adjustment (HR, 0.50; 95% CI, 0.30-0.84; p=0.008). The rate of stroke did not differ between the groups, with early hazard of the events observed in the surgically treated patients (HR, 1.17; 95% CI, 0.62-2.22; p=0.62). Detailed results of multivariate regression analysis for primary and secondary outcomes measures are provided in the supplementary materials together with Kaplan–Meier curves for the risk of death adjusted for EuroSCORE 2 scale components. (Supplementary Figures 1–5).

## DISCUSSION

The study presents a comparison of long-term outcomes of CABG and PCI in patients with multivessel CAD and HFrEF. The main findings are as follows: (1) the patients treated with PCI had worse clinical profile, whereas the surgical patients had more advanced atherosclerosis; (2) long-term survival rates were similar in both groups; (3) PCI was associated with increased risk of recurrent MI and urgent repeat revascularization in long-term observation.

CAD is the etiologic factor of two-thirds of all systolic HF cases and its treatment poses a significant challenge [10]. Even though revascularization has been shown to improve the prognosis, data on specific therapeutic strategies is scarce and mostly related to CABG [3]. In part, it is a consequence of former studies demonstrating a survival benefit of CABG over medical treatment in patients with reduced LVEF and multivessel disease [6]. There was no such comparison for PCI. However, PCI has been shown to exceed the number of CABG in this population, even despite the lack of compelling data from contemporary randomized studies [4, 11, 12]. This was also confirmed in our prior analysis [13]. Although reduced LVEF should guide the patient toward surgery, it seems that together with excessive morbidity it reduces the chance of CABG performance. Both significantly increase the risk of open heart surgery. On the other hand, patients with ischemic etiology of HF usually present high atherosclerosis burden in the coronary arteries. It is confirmed that in patients with advanced atherosclerosis reflected in high Syntax Score, survival benefit from CABG in comparison to PCI is more distinct and such patients should be treated surgically [7]. Results of our analysis were consistent with these observations. Patients treated with PCI had higher incidence of typical risk factors, prior ischemic events and more pronounced HF symptoms, while patients qualified for CABG had more complex atherosclerosis with higher occurrence of LM stenosis, triple-vessel disease and chronic total occlusions. Moreover, in the analyzed population, the overall frequency of neoplastic disease was higher in the PCI group. The presence of a neoplastic disease in CAD patients often drives the therapeutic decisions towards the less invasive modalities, to minimize the risks related to the cardiopulmonary bypass procedure [14]. Nonetheless, there were no significant differences between the study groups in the presence of history of malignant neoplasms. Additionally, complete revascularization was achieved more often in CABG patients, confirming that if surgery is possible, it offers more complex treatment.

There is scant evidence of PCI and CABG in HFrEF patients, because none of the completed revascularization randomized trials were focused on this population. Moreover, in the trials comparing these two revascularization strategies in stable conditions, patients with severe LV dysfunction where underrepresented or excluded [15]. In the recently published trials percentage of HFrEF patients was very low, 2% and 2.5% respectively, and it was also insufficient to reliably compare PCI and CABG in this population. [7, 8]. In two previous, relatively large trials, patients with decreased LV ejection fraction represented 22% and 21% of the study population. In the subgroup analysis, there were no differences in outcome between both methods, but PCI was performed with either balloon angioplasty or bare-metal stents and these analyses combined together consisted of less than 500 patients [16, 17]. Therefore, we must turn for guidance at observational studies. However, their heterogeneity makes them difficult to interpret. In two recent registries focused on HFrEF patients, only the minority of the patients had truly reduced LVEF [11, 18]. These data are difficult to extrapolate to the HF population with severely impaired LVEF. A direct comparison of PCI and CABG in the ischemic heart failure population was published recently by Bangalore et al., comparing 1063 matched pairs of patients treated either with PCI with everolimus-eluting stents or CABG. Overall rates of long-term all-cause mortality were similar in both groups. In the PCI group there were fewer strokes, but more myocardial infarctions and repeat revascularizations over a median follow-up of 2.9 years [19]. Although these results came from a large registry, there were some major exclusion criteria: LM disease, prior cardiac surgery, prior PCI. In our analysis, patients with prior cardiac surgery and/or prior percutaneous revascularization constituted one-third and two-thirds of patients in CABG and PCI arm respectively. Moreover, LM disease was found in 38% of CABG patients and in 13% of PCI patients. Therefore, almost half of our population would not have been included in the abovementioned analysis. This is probably the first report analyzing a wide HFrEF population almost without exclusions.

A diminishing mortality gap between CABG and PCI may be partially explained by the technological progress in interventional cardiology. New-generation thin-strut DES and additional devices allow for more complex interventions in more complex anatomy. Alongside improvements in the devices themselves, use of fractional flow reserve for functional revascularization, intravascular imaging for stenting deployment, periprocedural LV support in very high-risk subgroups or more potent antiplatelet drugs may ultimately influence the results. On the other hand, surgery addresses not only the current lesion, but also the disease that might progress and become a culprit in the future. In may be of importance in patients with HFrEF who will be less tolerant to repeated myocardial injury. In fact, in our population the risk of recurrent MI and urgent repeat revascularization was significantly higher in the PCI group.

In the present study, long-term survival after the adjustment for confounding factors was comparable for both revascularization strategies, while in the unadjusted population CABG offered significant reduction in mortality. Risk adjustment contains both clinical and angiographic parameters. This initial difference can be partly explained by worse clinical status and comorbidities among patients treated with PCI and more complex revascularization in the CABG patients.

These results come from an observational study and are only hypothesis-generating. However, for several years ischemic heart failure has been one of the major challenges in cardiovascular medicine and randomized trials in this field are still missing. The HFrEF population poses a substantial challenge in diagnosis and treatment. The majority of patients have multiple disorders, multifocal advanced atherosclerosis and numerous prior revascularization procedures. HF by itself is treated with sophisticated pharmacotherapy, comorbidities additionally increase the drug interaction. A possible combination of various factors creates an almost infinite number of clinical variants which should be addressed. One may speculate that attempts to perform a randomized clinical trial comparing CABG and PCI in HFrEF could be hampered by the inclusion criteria bias. Nowadays, all the available data should be incorporated to assist the decision-making process in routine clinical practice. In fact, very often a decision on revascularization in ischemic HF cannot be made solely based on current guidelines. A personalized approach towards every patient is mandatory, and therefore the role of the Heart Failure Team in this process is vital.

### Limitations

There are several limitations of our analysis. Firstly, due to observational design of the study, despite the use of advanced adjustment methods, potential selection biases could occur. Multivariate model analysis may also be biased because of a potential effect of confounding predictors that were not accessible. Secondly, we did not have data on anatomic SYNTAX risk score. Thirdly, in the PCI arm, the patients were treated both with BMS and DES, while DES are a gold standard now. Moreover, we did not assess cardiovascular mortality that may be of importance in this population.

## MATERIALS AND METHODS

### Patients selection

The patients were selected from the COMMIT-HF (COnteMporary Modalities in Treatment of Heart Failure) Registry and the Institutional Cardiac Surgery Database (ICSD). The COMMIT-HF registry has been described elsewhere [13]. In brief, it is a prospective ongoing registry of an all-comer systolic heart failure patient population (LVEF<=35%) treated in the Third Chair and Department of Cardiology since 2009. The ICSD is the institutional registry of cardiac surgery operations and covers all the procedures performed in the Silesian Center for Heart Diseases since 2006., Only the patients with confirmed multivessel CAD defined as severe stenosis (>=70%) in at least 2 major epicardial arteries or stenosis (≥50%) of the left main (LM) and severely reduced ejection fraction (LVEF≤35%), who underwent PCI or CABG were included into the analysis. Only the patients with stage C or D according to ACCF/AHA chronic heart failure classification were included into the analysis [20]. The patients with concomitant severe mitral valve regurgitation or severe aortic valve stenosis, myocardial infarction within 5 days preceding the index procedure, unstable hemodynamics or who were in cardiogenic shock, were excluded from the study. In both registries, LV ejection fraction was assessed by transthoracic echocardiography before the index procedure. Study design is shown in Figure 1. In the clinical characteristics, the presence of neoplastic disease has been analyzed based on the ICD-10 classification. Patients with C category at any time before revascularization were identified as having malignant neoplastic disease. This study has been granted permission from the Institutional Review Board and is consistent with the ethical standards laid down in the 1964 Declaration of Helsinki and its amendments.

### Interventions

Unfractionated heparin under the control of activated clotting time as well as P2Y12 inhibitor were administered to the patients undergoing coronary angioplasty. Balloon predilatation and postdilatation, the use of stents, type of stents, glycoprotein IIb/IIIa receptor inhibitors and others established interventional techniques were at the operator's discretion. In case of CABG, a decision on the extent and type of the procedure (classical CABG, off-pump CABG or minimally invasive direct coronary artery bypass) has been initially taken by the Heart Team and, if necessary, changed by the operators. In both groups, the treatment was performed with the intention to achieve complete revascularization (CR). For this analysis, an anatomic definition of CR was used. CR was defined as successful PCI of all angiographically significant lesions in all coronary arteries with a diameter of at least 2 mm, without a patent surgical graft in the PCI group, and grafting of all coronary arteries with a diameter of at least 2 mm and angiographically significant stenosis in the CABG group.

### Follow up

Both in-hospital and long-term follow-up were obtained in all the patients. In-hospital data came from the institutional electronic database which covered all clinical, angiographic, laboratory records and the history of hospitalization. All the adverse events were verified with source documentation. Data on patients from both registries were linked with the national health care provider database which covers all the hospitalizations and performed procedures after discharge. Data on mortality was obtained from the same source [21].

### Outcomes

Both short-term (30 days) and long-term prognoses were evaluated. The primary outcome of the study was long-term all-cause death. The secondary outcomes were myocardial infarction (MI), stroke and urgent repeat revascularization. Non-fatal MI was defined as an ischemic event that met the European Society of Cardiology/American College of Cardiology/American Heart Association criteria for myocardial infarction [22]. Repeat urgent revascularization was defined as an unplanned PCI or CABG, performed as urgent procedure due to acute ischemic symptoms. Stroke was defined as an ischemic event that is in accordance with European Stroke Organization guidelines [23].

### Statistical analysis

Variables were presented in tables containing the arithmetic mean with standard deviation for quantitative and frequency for qualitative parameters. Distribution of quantitative variables was tested by the Shapiro-Wilk test. Due to other than normal distributions, the verification of hypothesis of the parameters equality in individual groups was performed by the Mann—Whitney U test. Distribution of qualitative variables was evaluated using the Chi-squared test. The Kaplan–Meier estimator and the Cox Proportional Hazards Model were used to analyze long-term absolute survival and event-free survival. In order to assess the effect of the performed treatment on the outcome, a univariate model was calculated for each endpoint, containing only the information about the type of performed treatment. In the next step, the multivariable model containing parameters from baseline characteristics was generated.

Because of the differences in baseline characteristics of the analyzed groups, Propensity Score Based Adjusted Survival Curves were used [24]. Propensity score was calculated for the same variables used in Cox's multivariate analysis—the dependent variable was the mode of treatment. All the hypotheses were assumed as two-tailed-verified by two-tailed tests. For the purposes of the analysis, p-value <0.05 was considered as statistically significant. The R version 3.3.3 (2017-03-06) The R Foundation for Statistical Computing was used for all the calculations.

## CONCLUSIONS

Among the patients with HFrEF and multivessel coronary artery disease, similar rates of survival in the patients treated with either PCI or CABG were noted. PCI was associated with an increased risk of recurrent MI and urgent repeat revascularization, whereas the risk of stroke was similar for both methods. The results are hypothesis-generating and should be tested in prospective, randomized trials.

## SUPPLEMENTARY MATERIALS FIGURES AND TABLES



## Abbreviations

CABG: coronary artery bypass grafting
CAD: coronary artery disease
CI: confidence interval
CR: complete revascularization
HF: heart failure
HFrEF: heart failure with reduced ejection fraction
HR: hazard ratio
ICD-10: International Statistical Classification of Diseases and Related Health Problems
LM: left main
LV: left ventricle
LVEF: left ventricle ejection fraction
MI: myocardial infarction
PCI: percutaneous coronary intervention.
