# Supplementary material for: Comparison of coronary artery bypass grafting and percutaneous coronary intervention in patients with heart failure with reduced ejection fraction and multivessel coronary artery disease
Source: Oncotarget. 2018 Apr 20;9(30):21201–10. doi: 10.18632/oncotarget.25006 (PMC5940397; doi:10.18632/oncotarget.25006)
Supplement: Supplementary file 1 [file oncotarget-09-21201-s001.pdf]

Comparison of coronary artery bypass grafting and percutaneous coronary intervention in patients with heart failure with reduced ejection fraction and multivessel coronary artery disease

SUPPLEMENTARY MATERIALS

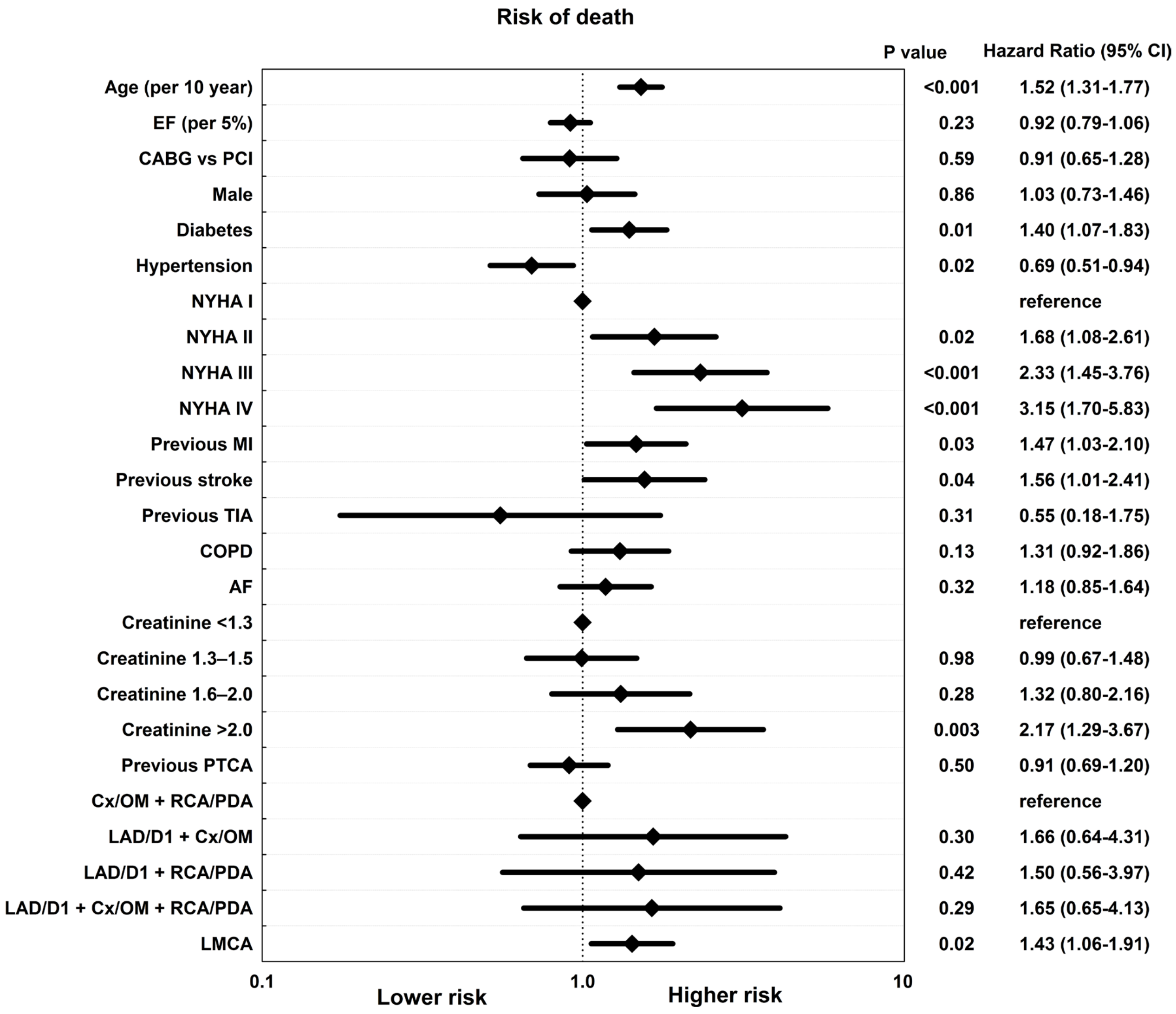

Supplementary Figure 1: Results of multivariate regression analysis for the risk of all cause death. AF – atrial fibrillation; CABG – coronary artery bypass grafting; CI – confidential interval; COPD – chronic obstructive pulmonary disease; Cx – circumflex artery; D1 – first diagonal artery; EF – ejection fraction; IM – intermediate branch; MI – myocardial infarction; NYHA - New York Heart Association classification; LAD – left anterior descending; LMCA – left main coronary artery; OM – obtus marginal branch; PCI – percutaneous coronary interventions; PDA – posterior descending artery; PTCA - percutaneous transcatheter coronary interventions; TIA – transient ischemic attack; ; RCA – right coronary artery.

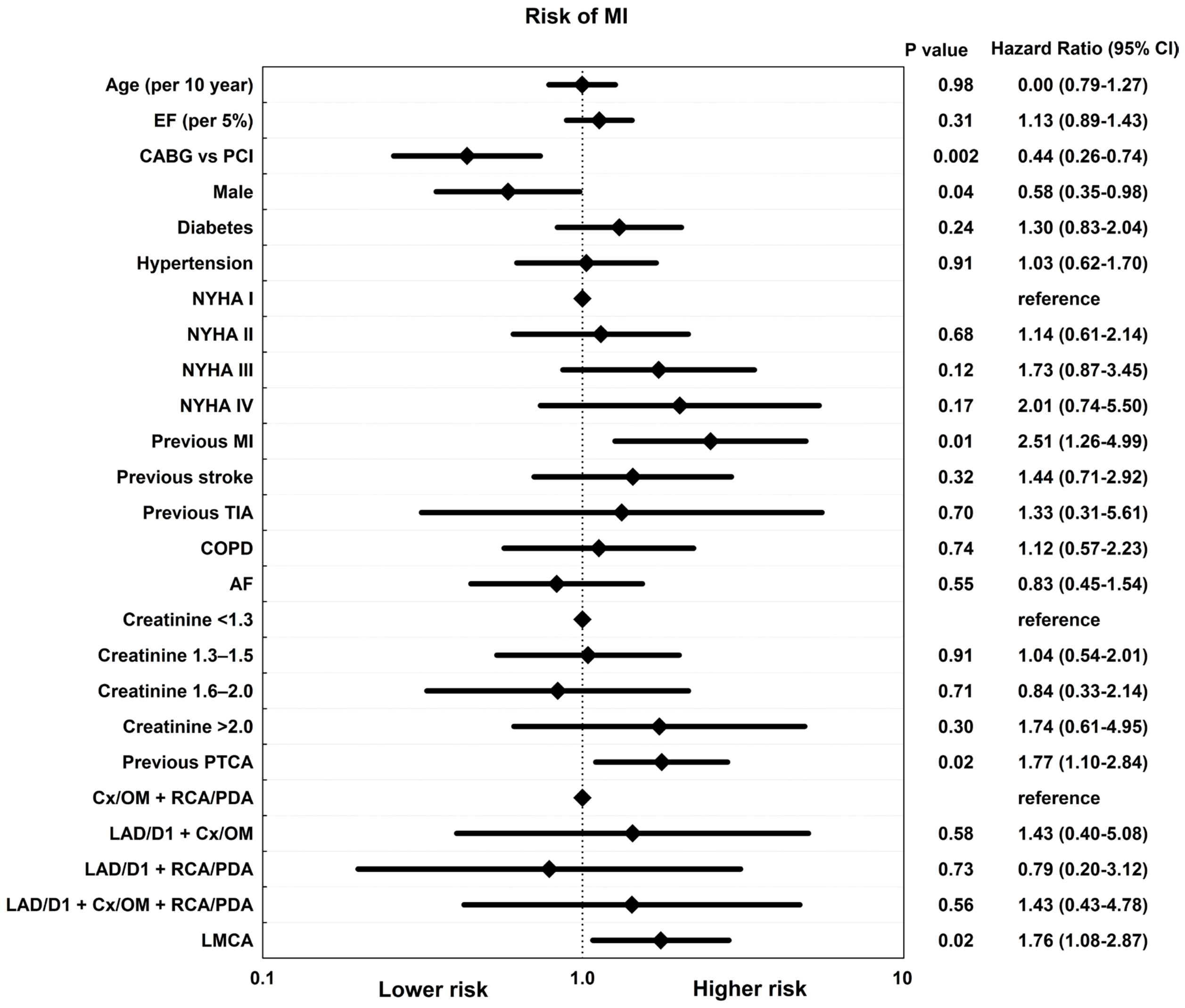

Supplementary Figure 2: Results of multivariate regression analysis for the risk of myocardial infarction. AF – atrial fibrillation; CABG – coronary artery bypass grafting; CI – confidential interval; COPD – chronic obstructive pulmonary disease; Cx – circumflex artery; D1 – first diagonal artery; EF – ejection fraction; IM – intermediate branch; MI – myocardial infarction; NYHA - New York Heart Association classification; LAD – left anterior descending; LMCA – left main coronary artery; OM – obtus marginal branch; PCI – percutaneous coronary interventions; PDA – posterior descending artery; PTCA - percutaneous transcatheter coronary interventions; TIA – transient ischemic attack; ; RCA – right coronary artery.

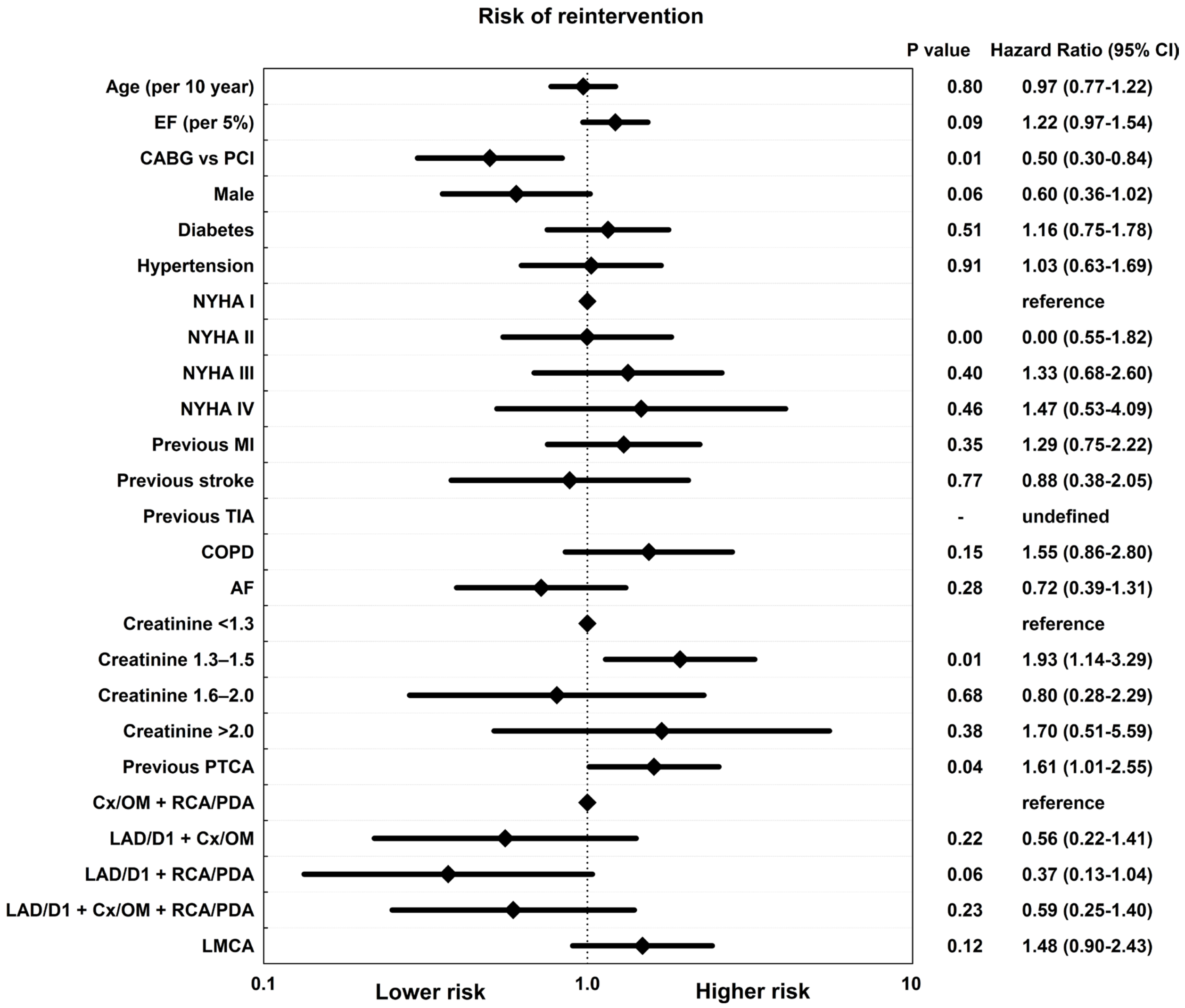

Supplementary Figure 3: Results of multivariate regression analysis for the risk of repeat urgent revascularization. AF – atrial fibrillation; CABG – coronary artery bypass grafting; CI – confidential interval; COPD – chronic obstructive pulmonary disease; Cx – circumflex artery; D1 – first diagonal artery; EF – ejection fraction; IM – intermediate branch; MI – myocardial infarction; NYHA - New York Heart Association classification; LAD – left anterior descending; LMCA – left main coronary artery; OM – obtus marginal branch; PCI – percutaneous coronary interventions; PDA – posterior descending artery; PTCA - percutaneous transcatheter coronary interventions; TIA – transient ischemic attack; ; RCA – right coronary artery.

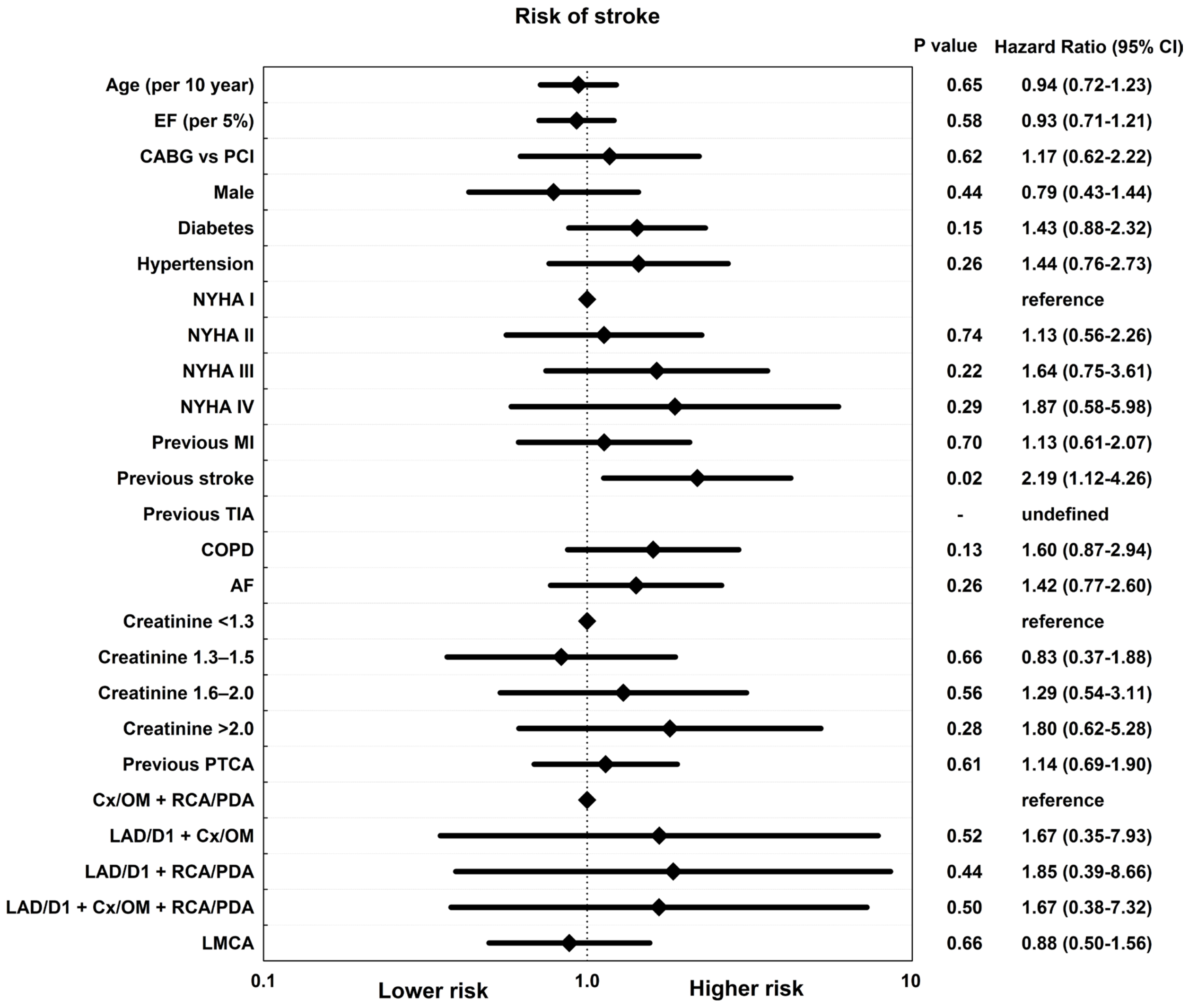

Supplementary Figure 4: Results of multivariate regression analysis for the risk of stroke. AF – atrial fibrillation; CABG – coronary artery bypass grafting; CI – confidential interval; COPD – chronic obstructive pulmonary disease; Cx – circumflex artery; D1 – first diagonal artery; EF – ejection fraction; IM – intermediate branch; MI – myocardial infarction; NYHA - New York Heart Association classification; LAD – left anterior descending; LMCA – left main coronary artery; OM – obtus marginal branch; PCI – percutaneous coronary interventions; PDA – posterior descending artery; PTCA - percutaneous transcatheter coronary interventions; TIA – transient ischemic attack; ; RCA – right coronary artery.

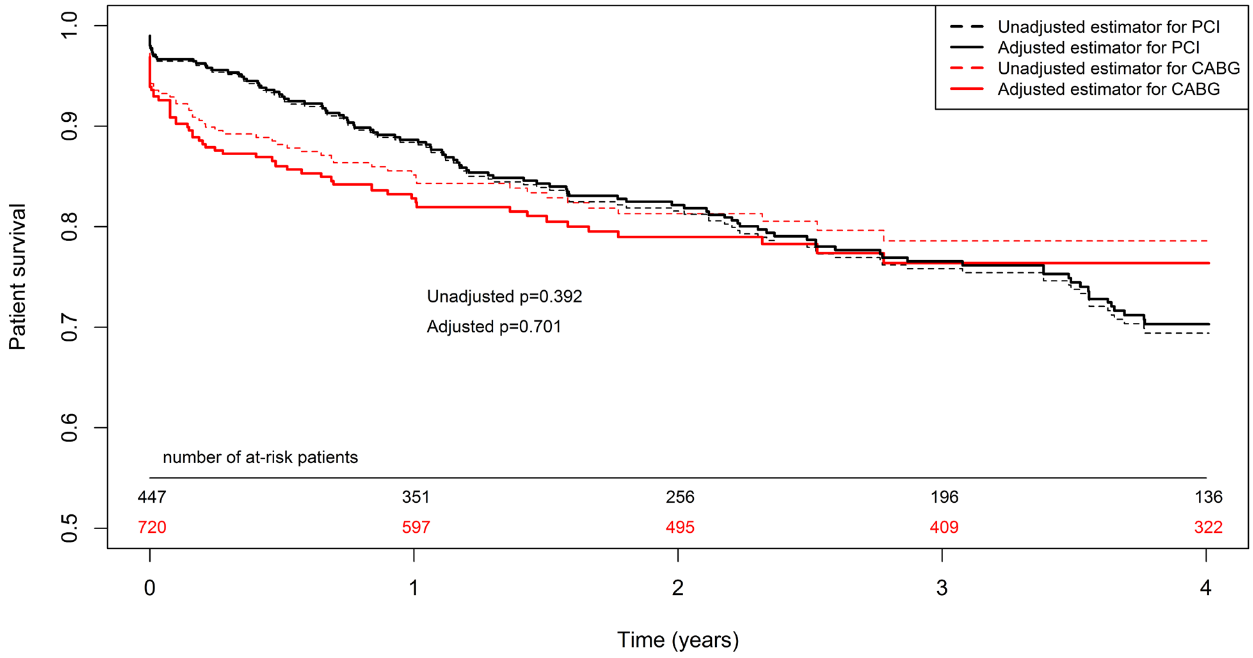

Supplementary Figure 5: Kaplan-Meier curves and Forest plot for long term all-cause death adjusted for Euroscore 2 scale components. CABG – coronary artery bypass grafting; PCI – percutaneous coronary interventions; Results adjusted for EuroSCORE 2 scale components.

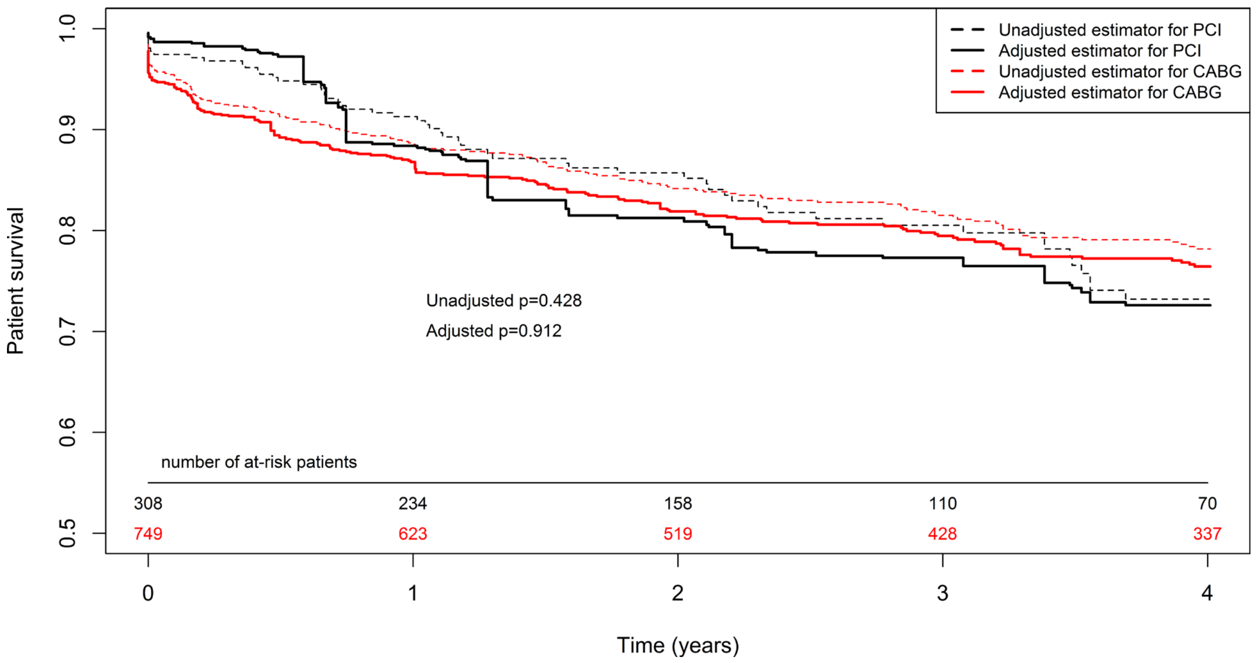

Supplementary Figure 6: Kaplan-Meier curves for long term all-cause death. Results adjusted for: sex, age, diabetes mellitus, hypertension, New York Heart Association classification at admission, Left Ventricle Ejection Fraction, previous stroke, previous transient ischaemic attack, chronic obstructive pulmonary disease, atrial fibrillation, chronic kidney disease, previous PCI, left main disease, two- and three vessel coronary artery disease.

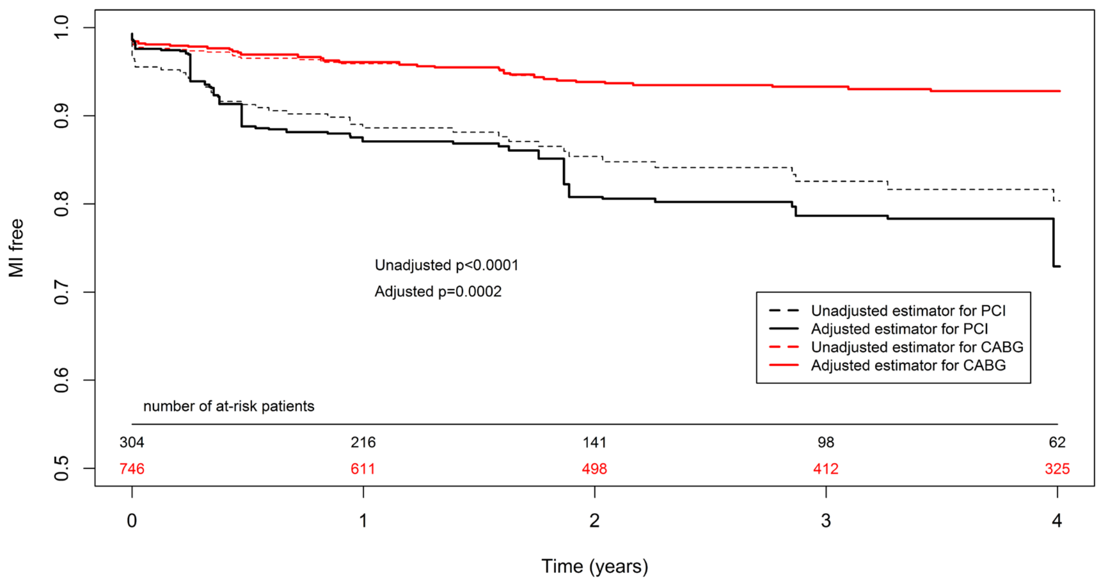

Supplementary Figure 7: Kaplan-Meier curves for long term myocardial infarction. Results adjusted for: sex, age, diabetes mellitus, hypertension, New York Heart Association classification at admission, Left Ventricle Ejection Fraction, previous stroke, previous transient ischaemic attack, chronic obstructive pulmonary disease, atrial fibrillation, chronic kidney disease, previous PCI, left main disease, two- and three vessel coronary artery disease.

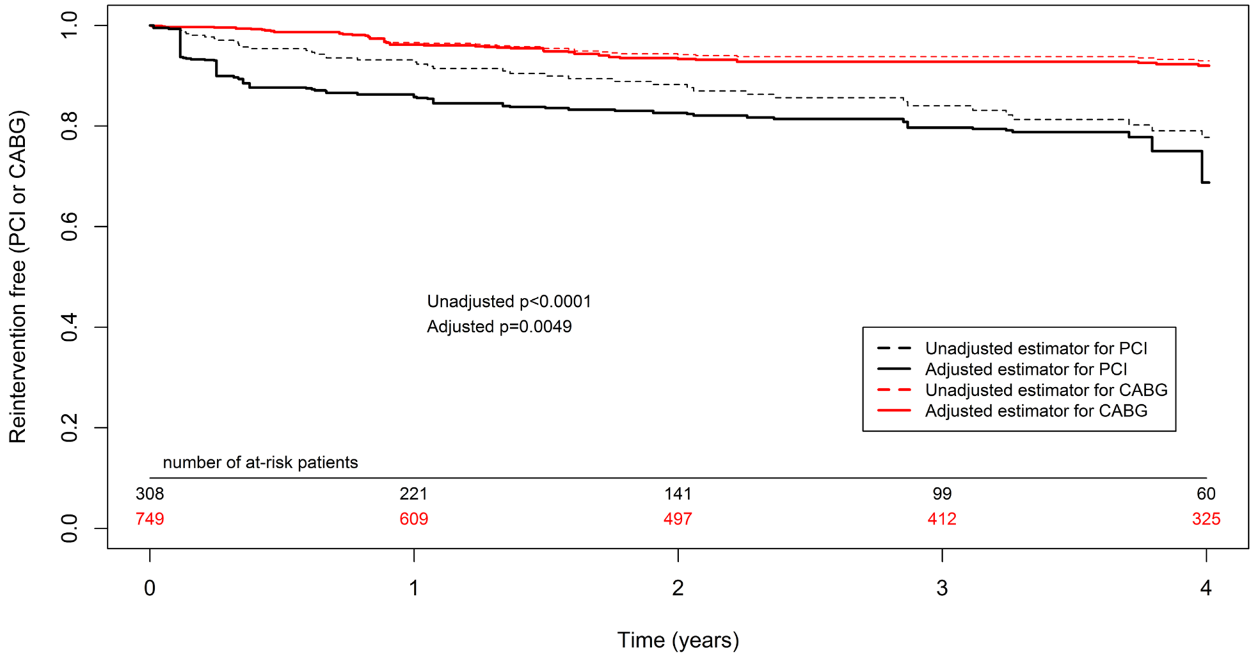

Supplementary Figure 8: Kaplan-Meier curves for long term repeat urgent revascularization. Results adjusted for: sex, age, diabetes mellitus, hypertension, New York Heart Association classification at admission, Left Ventricle Ejection Fraction, previous stroke, previous transient ischaemic attack, chronic obstructive pulmonary disease, atrial fibrillation, chronic kidney disease, previous PCI, left main disease, two- and three vessel coronary artery disease.

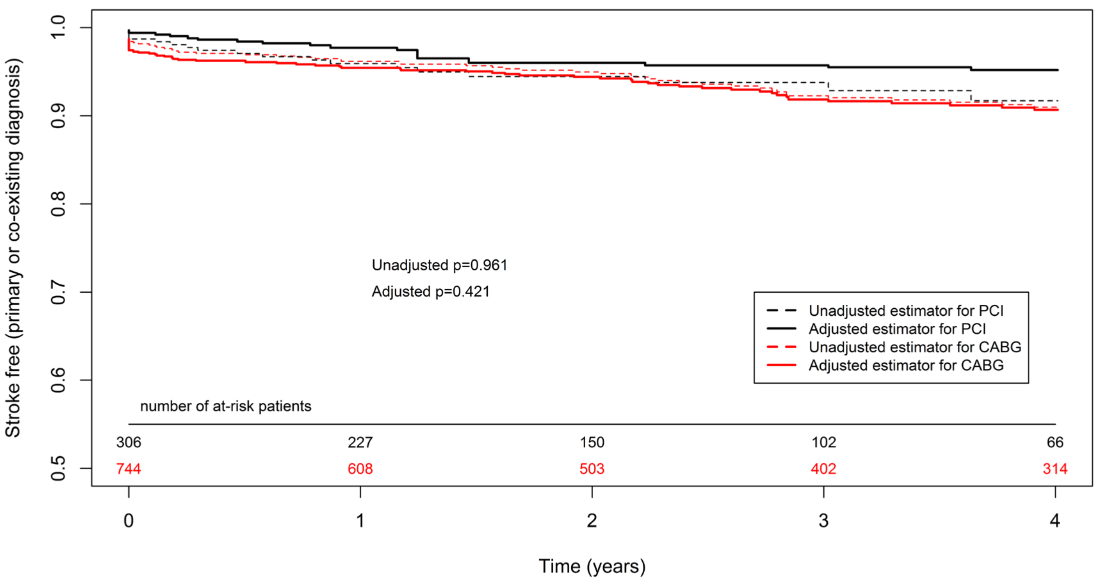

Supplementary Figure 9: Kaplan-Meier curves for long term stroke. Results adjusted for: sex, age, diabetes mellitus, hypertension, New York Heart Association classification at admission, Left Ventricle Ejection Fraction, previous stroke, previous transient ischaemic attack, chronic obstructive pulmonary disease, atrial fibrillation, chronic kidney disease, previous PCI, left main disease, two- and three vessel coronary artery disease.

Supplementary Table 1: Baseline characteristics

Factor	Study population	N = 1072	P value
	CABG N = 761	PCI N = 311	
Age, years ± SD	64.7 ± 9.0	64.7 ± 9.7	0.86
Male, %	82.5	86.5	0.13
BMI, kg/m^2 ± SD	27.7 ± 4.4	28.7 ± 44.26	0.006
Arterial hypertension, %	79.2	63.3	<0.001
Prior one MI, %	54.9	48.9	0.08
Prior two or more MI, %	20.6	19.0	0.06
Prior PCI, %	37.6	64.6	<0.001
Prior CABG, %	0.5	24.8	<0.001
Atrial fibrillation, %	11.6	22.5	<0.001
Prior stroke, %	9.1	7.0	0.34
Diabetes mellitus, %	37.3	46.0	<0.001
Dyslipidemia, %	62.0	46.9	<0.001
COPD, %	13.4	8.4	0.03
Neoplastic disease, %	16.5	20.9	0.01
Malignant neoplasms, %	9.5	12.9	0.03
Benign neoplasms, %	7.0	8.1	0.33
NYHA Class*			
I, %	21.3	19.6	0.59
II, %	59.9	38.3	<0.001
III, %	17.1	34.7	<0.001
IV, %	1.7	7.1	<0.001
eGFR*, 30-60 ml/min/1.73m^2, %	11.3	12.2	0.74
eGFR*, < 30 ml/min/1.73m^2, %	7.4	8.1	0. 31
LVEF*, % ± SD	30.9 ± 4.5	27.1 ± 5.4	<0.001
EUROSCORE 2 scale, %	3.64 ± 4.36	5.14 ± 4.86	<0.001

BMI - body mass index; CABG - coronary artery bypass grafting; CAD - coronary artery disease; COPD - chronic obstructive pulmonary disease; eGFR = estimated glomerular filtration rate; LVEF - left ventricular ejection fraction; MI - myocardial infarction; NYHA – New York Heart Association; PCI - percutaneous coronary intervention; Q1-Q3 - quartile 1 and quartile 3; SD - standard deviation.

Supplementary Table 2: Angiographic and procedural characteristics

Factor	Study population	N = 1213	P value
	CABG N = 761	PCI N = 311	
2-vessel disease, %	24.8	46.3	<0.001
3-vessel disease, %	75.2	53.7	<0.001
Territory			
LM, %	38.5	15.8	<0.001
LAD/D1, %	99.5	92.3	<0.001
Cx/OM/IM, %	91.7	81.0	<0.001
RCA/PDA, %	84.0	80.4	0.18
CTO, %	56.5	40.2	<0.001
CTO, mean ± SD	0.85 ± 0.91	0.51 ± 0.71	<0.001
No of grafts, mean ± SD	2.50 ± 0.93		
- Arterial grafts, mean ± SD	1.01 ± 0.50		
- Saphenous grafts, mean ± SD	1.49 ± 0.97		
No of stents DES, mean ± SD		1.29 ± 1.19	
Complete revascularization, %	68.6	54.0	<0.001

CABG - coronary artery bypass grafting; CTO – chronic total occlusion; Cx – circumflex artery; D1 – first diagonal branch; DES – drug eluting stents; IM – intermediate branch; LAD – left anterior descending; LM – left main; OM – obtus marginal branch; PDA – posterior descending artery; PCI – percutaneous coronary intervention; RCA – right coronary artery; SD - standard deviation.

Supplementary Table 3: In-hospital, 30 day and 1 year outcomes

Factor	Study population	N = 1213	P value
	CABG N = 761	PCI N = 311	
In-hospital			
death, %	1.6	1.0	0.62
MI, %	0.9	1.6	0.51
stroke, %	0.8	0.6	0.88
30 day			
Death, %	3.0	1.6	0.26
MI, %	1.5	2.9	0.18
Stroke, %	1.1	0.6	0.77
1 year			
Death, %	9.7	6.8	0.20
MI, %	2.0	7.5	0.001
Stoke, %	2.3	2.7	0.85

CABG – coronary artery bypass grafting; MI – myocardial infarction; PCI – percutaneus coronary intervention.
